# Early Questing by Lone Star Tick Larvae, New York and Massachusetts, USA, 2018

**DOI:** 10.3201/eid2508.181293

**Published:** 2019-08

**Authors:** Sam R. Telford, Joanna Buchthal, Paul Elias

**Affiliations:** Tufts University, North Grafton, Massachusetts, USA (S.R. Telford III);; Massachusetts Institute of Technology, Cambridge, Massachusetts, USA (J. Buchthal);; Naushon Trust, Boston, Massachusetts, USA (P. Elias)

**Keywords:** ticks, host-seeking, Lone Star tick, phenology, parasites, New England, New York, Massachusetts, United States, vector-borne infections, zoonoses

## Abstract

Subtropical lone star tick larvae typically emerge in late summer. We found clusters of host-seeking lone star tick larvae during early June 2018 in New York and Massachusetts, USA. Invasion and persistence of this tick in more northern locations may have been promoted by adaptation to an accelerated life cycle.

Lone star ticks (*Amblyomma americanum*) transmit diverse agents of zoonoses, including human monocytic ehrlichiosis, canine granulocytic ehrlichiosis, Rocky Mountain spotted fever, and tularemia. Their bites also may induce erythema migrans, for which the etiology remains elusive (*1*) and which seems to be the cause of red meat (alpha-gal [galactose-α-1,3-galactose]) allergy. 

The distribution and abundance of these ticks in the United States have recently expanded; specimens have been collected from the upper Midwest and New England (*2*,*3*). However, proof of stable infestations at most of these locations remains to be published. Before the 1990s, the known northern limit for dense infestations in the eastern United States was New Jersey (*4*), although since the late 1980s, a relict stable population of lone star ticks has been present on Prudence Island, Rhode Island (*5*), and on Fire Island, eastern Long Island, New York (*6*). Unlike the ticks on Long Island, those on Prudence Island have remained confined there, with no spread to the nearby mainland. Models suggest that climate change could facilitate the introduction of this subtropical tick into more northern locations (*3*) because temperature and relative humidity are the main drivers for the developmental cycle. In the past 5 years, we have found focal persisting lone star tick infestations (documentation of larvae, nymphs, and adults each year for 3 years) on Cape Cod, the Elizabeth Islands, Martha’s Vineyard, and Tuckernuck Island (all in Massachusetts), which, to our knowledge, is the northernmost established population of this tick.

Across their wide distribution south of New York, adult lone star ticks seek hosts mid-March through late June; nymphs, mid-May through late July; and larvae, July through September (*7*). Fed larvae and nymphs overwinter and molt to nymphs and adults, respectively, the following spring. Of note, female engorgement may be suppressed until mid-May, perhaps associated with a photoperiodically regulated diapause (*8*), which would enable egg masses to be deposited during optimal temperatures and humidity. Fed females oviposit soon after engorgement, and in most locations, resulting larvae seek hosts from July through September. A comprehensive simulation model based on existing reports of lone star tick phenology (*7*) suggested a threshold of 17°C for larval host seeking. On Long Island and in New Jersey, larvae were most commonly found in August (*4*,*6*). Lone star tick larvae seem to feed mainly during late summer and early fall.

During standard drag sampling for deer tick (*Ixodes dammini*) nymphs on June 7, 2018, in New Suffolk, Long Island, New York, and on June 18, 2018, on Naushon Island, Massachusetts, we identified well-defined clusters (≈6 cm diameter) of unusual host-seeking tick larvae. Three clusters were found during 10 person-hours of dragging at the New York site and 2 clusters during 6 hours of dragging at the Massachusetts site; all clusters contained >100 larvae. Lone star tick adults and nymphs accounted for >95% of all ticks collected from these sites; the rest were deer tick nymphs and American dog tick (*Dermacentor variabilis*) adults. The clusters of larvae (Figure, panel A) were reminiscent of those of lone star ticks typically encountered during August or September on Prudence Island, but tick collection there on June 4, 2018 (9 person-hours), where these ticks have long been endemic (*5*), did not find any such clusters. Samples of larvae from New Suffolk and Naushon Island were collected on masking tape lint rollers for definitive identification. We removed larvae from the masking tape and examined them by microscopy at ×100 magnification. The presence of 11 festoons, long palpi, rounded lateral basis capitulum, and the general circular body morphology (Figure, panel B) confirmed that the samples were larval lone star ticks and not any other ticks that are common in southern New England, including deer ticks, American dog ticks (the subadults of which cannot be collected by drag sampling), winter ticks (*Dermacentor albipictus*), *Ixodes dentatus* ticks, and rabbit ticks (*Haemaphysalis leporispalustris*).

**Figure:**
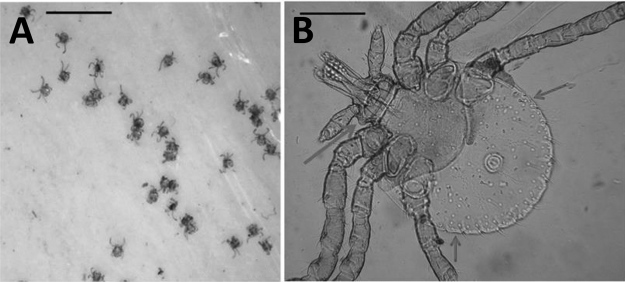
Larvae of lone star ticks collected from New York and Massachusetts, USA, in 2018. A) Portion of lint roller tape with cluster of larval lone star ticks. Scale bar indicates 10 mm. B) Rounded idiosoma, 11 festoons (short dark arrow marks festoon 1; longer dark arrow, festoon 11); rounded edge of the basis capitulum (long light arrow), and palps longer than wide. Scale bar indicates 210 microns.

Larval lone star ticks are typically found seeking hosts only in late summer. We are aware of only 1 report of early larval activity, in which infestation of white-footed mice (*Peromyscus leucopus*, a very unusual host for this tick) was detected in March and June at a New Jersey site (*4*). The Prudence Island tick population has been studied since the early 1990s (*9*), with sampling every year during June and other months, and host-seeking lone star tick larvae have not been found before August. The recent expanded range of lone star ticks into sites previously considered too cold to allow for the developmental cycle may be partly because of a new tick lineage that may develop unusually rapidly. The biological basis for the expanding range of this invasive vector species accordingly may include selection (*10*) for a more plastic phenology and for increasingly permissive weather at the edge of its range. This fundamental change may facilitate the invasion and establishment of an aggressive human-biting disease vector, which may increase risk for diverse tickborne infections. 
